# The Council on Pharmacy and Chemistry, Present and Future

**Published:** 1919-06

**Authors:** W. A. Puckner

**Affiliations:** Secretary, Council on Pharmacy and Chemistry, American Medical Association


### THE COUNCIL ON PHARMACY AND CHEMISTRY, PRESENT AND FUTURE

W. A. PUCKNER, PHAR.D., SECRETARY, COUNCIL ON PHARMACY AND CHEMISTRY, AMERICAN MEDICAL ASSOCIATION

Organized primarily for the purpose of putting a stop to false declarations with regard to the composition of proprietary medicines, the Council's activities have broadened until its work may be characterized as "a propaganda for the rational use of drugs." The following are some of its activities:

1. *New and Nonofficial Remedies.*—This is an annual volume, issued by the Council. It describes both proprietary and unofficial nonproprietary drugs which are deemed worthy of consideration by the medical profession. To be admitted to this book, a preparation must comply with certain definite rules which stipulate, in effect, that its composition be declared, that no untrue or grossly exaggerated claims be made for it, and that it shall give promise of having therapeutic value.

With the exception of a few which are still under consideration, the Council has considered all proprietaries whose owners or accredited agents have requested that an examination of the products be made, and it has admitted to the book those which were found eligible. In addition, the Council has examined all of the more important or widely exploited proprietaries, even when no examination was requested, and it has admitted those of this group which were found eligible. Further, the Council has admitted to the book certain unofficial, nonproprietary articles which seemed to give promise of therapeutic usefulness, and it has established standards for the control of their identity and purity, and listed those brands which complied with these standards.

As most proprietary medicines are of a more or less experimental nature, they are accepted for inclusion in "New and Nonofficial Remedies" only for a limited time—usually a period of three years. At the expiration of the period of acceptance, each preparation is reexamined and retained only if the claims made for it and the present day knowledge of its value permit this action.

Since manufacturers gave information only in regard to their own products, "New and Nonofficial Remedies" groups together articles of a similar character, and includes in each case a general discussion of the group for the purpose of comparison, not only with each other, but also with the established or pharmacopoeial drugs which members of the group are intended to supplant.

In brief, "New and Nonofficial Remedies" is a book in which are described preparations that have been accepted by the Council. The description includes facts that the physician should have. It is a book that should be in the hands of every physician who prescribes medicines, and who wishes to know the facts regarding the newest remedies. It is the only book in which he can find information relative to proprietary medicines that are worthy of his patronage. It will protect the physician who makes use of it against the wiles of the promoters of products not worthy of his patronage. It would certainly be of use to the physician when the detail man calls on him, for if he were being importuned to prescribe or use samples of something which he had not heretofore used and which he was unable to find in "N. N. R.," he might ask the detail man why. In the nature of things few physicians are sufficiently expert in chemistry and allied sciences to be able unerringly to discriminate between the true and the false as regards many preparations that he is asked to prescribe.

2. *The Reports of the Council on Pharmacy and Chemistry.*—A medicament may be inadmissible to "New and Nonofficial Remedies" for various reasons: it may be worthless or irrational, its composition may be secret or indefinite, or it may be exploited under exaggerated or unwarranted claims or in a way otherwise detrimental to the public health and scientific medicine. Of these various reasons which make an article unacceptable, the manufacturer obviously may remove all except the first, viz., worthlessness or irrationality. Consequently, a preparation which has been presented for admission is not definitely rejected until after its proprietor has been informed of the objections to his product and has failed to bring the preparation in conformity with the Council's rules. When a preparation is found definitely inadmissible to "New and Nonofficial Remedies," that is, when the proprietor can not or will not make it acceptable, the Council prepares a report for publication. These reports are sent for publication to *The Journal of the American Medical Association*, and later published in the "Annual Reports of the Council on Pharmacy and Chemistry." The more important of these are also published in the book, "The Propaganda for Reform in Proprietary Medicines."

3. *Useful Drugs.*—Since the domination of proprietary medicines, which was retarding medical advance and threatening therapeutic chaos, had been made possible only by the insufficient and inefficient instruction given in medical schools in subjects having to do with drugs, the Council appointed a "Committee on Medical Teaching" to secure the cooperation of teachers in *materia medica*, pharmacology and related branches. This committee has endeavored to effect an improvement in these courses of instruction. One of the results of this work was the selection of a list of drugs to serve as a basis of *materia medica* instruction and thus insure that medical students shall be better informed with regard to the therapeutic worth of a few well established drugs, rather than, as in the past, leaving school with a smattering of knowledge about many drugs. The outcome of these efforts is the publication of "Useful Drugs," a concise but thorough and up-to-date discussion of the actions, uses and dosage of the more important drugs. The list of drugs presented in this book is now the basis of instruction in many schools; and many state examining boards are confining their *materia medica* questions to the drugs in the list.

4. *Epitome of the U. S. P. and N. F.*—To encourage the use of official drugs and to make available an estimate of their therapeutic value, a committee of the Council prepared an abstract of the U. S. Pharmacopoeia and the National Formulary. This booklet, the "Epitome of the U. S. Pharmacopoeia and National Formulary," presents those portions of these books which are of interest to physicians, and in addition, gives a concise statement of the therapeutic usefulness of the drugs and preparations described in them.

5. *Patent Law Reform.*—Some of the worst abuses connected with the exploitation of proprietary medicines have been made possible by our patent and trademark laws and the method of their interpretation and enforcement. The Council therefore appointed a committee to study these laws and the various propositions advanced for their improvement. This committee has published, from time to time, reports on various phases of our patent and trademark laws and recently summarized these reports in an address sent to the commissioner of patents and the interested congressional committees. It is hoped that by means of these reports physicians will be enabled to give intelligent support to a revision of the patent and trademark laws when legislation is proposed.

6. *Therapeutic Research.*—Through its Committee on Therapeutic Research, and with the aid of funds provided by the Board of Trustees of the American Medical Association, the Council has encouraged the investigations of questions which might lead to a better understanding of the action of drugs. These investigations are brought together in the annual reports of the Therapeutic Research Committee of the Council, and are an important addition to our knowledge of drug action.

In the past, the Council has in particular encouraged the investigation of the action and therapeutic value of widely used drugs regarding which our knowledge is still unsatisfactory. These investigations have included a study of the action of strychnine in cardiac disease, a comparison of the action of absorption and excretion of iodide preparations, a study of the pharmacology of the opium alkaloids, etc. Appreciating that the available knowledge of proprietary drugs is one sided in that it comes from investigations made by interested pharmaceutical concerns or from investigations made at the instigation of these firms, the Council is planning a comprehensive study of many of the synthetic drugs that have gained some vogue during recent years.

### THE FUTURE

Medical research, and efficient instruction in therapeutics and related subjects, spell a diminishing influence of commercial medicine over rational therapeutics. The fact that the present shortage of German synthetics has not handicapped seriously the practice of medicine should be a lesson to American physicians for many years to come.

On the other hand, it must be remembered that the publicity given to the reports of the Council and to other contributions toward rational therapeutics by *The Journal of the American Medical Association*, the journals of the state organizations, and a few personally owned publications, is as nothing when compared with the persistent and wide publicity given to the propaganda of the proprietary houses. While a report setting forth the objections to a proprietary is published but once, the firm's laudatory pronouncement goes forth again and again until the Council's report is completely overwhelmed and forgotten.

Not only do manufacturers of proprietaries keep in close touch with the practicing physician by means of house organs, special "literature," or by traveling representatives, but many of the firms, through the meritorious lines of pills, tablets, tinctures, etc., which they put out, obtain and hold the good will and confidence of a large proportion of the medical profession.

Furthermore, some of these firms not only gain the confidence of the medical profession through these high-grade pharmaceuticals, but certain of their proprietaries may be of distinct therapeutic value and yet fail to be acceptable for "New and Nonofficial Remedies," because they do not conform with the reasonable rules of the Council. These firms do not find it profitable to force the sale of their regular nonproprietary pharmaceuticals by unwarranted claims or objectionable methods, yet they may consider it good business to market certain proprietary products by means of claims which are extravagant and without warrant, and which will lead to indiscriminate use by the profession and the public. In a word, where there is one dollar spent on behalf of rational medicine, thousands are spent for the purpose of increasing the sale of preparations which directly or indirectly are a detriment to the public health, to medicine, and to the pocketbook.

Progress toward a rational and scientific drug therapy must continue, and, therefore, it is important that the Council on Pharmacy and Chemistry should continue to make the investigation of proprietary medicines its chief work. Investigation of a proprietary medicine, however, and a report of such investigation are of value in direct ratio only to the number of physicians who read the report, endorse it and act in accordance with its conclusions. In order that you may determine to what extent those preparations which are admitted to "New and Nonofficial Remedies" deserve your interest and confidence, it will be worth while briefly to outline the rules which govern the

Council in the admission of articles to "New and Nonofficial Remedies."

RULES GOVERNING THE ACCEPTANCE OF ARTICLES FOR "N. N. R."

*Composition.*—Rules 1 and 2, and in a measure 5, 7, and 9, deal with the composition of articles. Rule 1 requires that the quantative composition of an article be furnished the Council for publication. Rule 2 requires that the manufacturer furnish methods whereby the composition of products that are definite chemicals or the potent constituents of mixtures may be determined. The Council does not require that the process of manufacture of an article be declared unless this becomes necessary in order to judge its composition. Rule 5 requires that statements with regard to the origin and source of an article shall be truthful. Rule 7 requires that for the guidance of the dispenser, the amounts of poisonous ingredients of a preparation be placed on the label. Rule 9 requires that if patent rights are claimed for a product, the Council be informed on this point.

That it is not only the right but also the duty of the physician to know the composition of what he prescribes for his patients is so generally admitted that few have attempted to market preparations of avowedly secret composition. When the Council first began its work, it was common to see chemical formulas or statements of composition published which a chemist or a pharmacist was able to pronounce at a glance as impossible. It was not unusual to find that the promoter published "a formula" for his preparation, rather than "the formula." Today, however, a more prudent, if not more honest, course is pursued. This gives a "formula" which is correct so far as it goes, but which fails to divulge the actual composition of a preparation. When it is considered that many physicians are not any too conversant with the chemistry and pharmacy of drugs, it is not surprising that some administered the proprietary "Venarsen," regarding the composition of which they had only the vague statement that it was ". . . . a comparatively nontoxic, organic, arsenic compound, 0.6 gm., representing 247 mg. (3¾ grains) of metallis arsenic in chemical combination . . ." in the belief that a preparation similar to that first introduced as salvarsan was being used. That "Venarsen" contained its arsenic as sodium cacodylate—a notoriously inactive state of combination—does not justify the intravenous administration of a drug of unknown composition.

While for the present it probably is not feasible to require, on the part of those who manufacture medicinal preparations, such professional training as is required of those who prescribe and those who dispense them, it certainly is not too much to require, as does Rule 2, that a manufacturer shall be able to demonstrate that his preparation has the composition claimed for it.

The issuance of a patent for a medicinal product does not prove that such a product presents a discovery or that its owner is entitled to a temporary monopoly, yet it is only fair to physicians and to other manufacturers that notice of such patent claims be given. Hence, the Council publishes in "New and Nonofficial Remedies" the information bearing on this point.

*Lay Advertising.*—Rules 3 and 4 provide against the recognition of articles that are advertised to the public directly or indirectly, exempting from this requirement preparations which the Council believes are safe to be so advertised.

It has been held with some justice that certain shotgun proprietaries are purchased by the public with as much circumspection as they are ordered by those physicians who are addicted to the prescribing of them; but even the exploiters of these mixtures have not denied that the use of medicines by the public on its own initiative is surrounded with many objections. Hence the practice of self medication should not be encouraged by prescribing or using those preparations advertised for public use.

The only objection to the rule has come from a firm which markets a brand of liquid petrolatum. The Council has considered the question of exempting simple laxatives from the restrictions of Rules 3 and 4, as it has exempted antiseptics and nonmedicinal foods. The conclusion was, however, that the excessive use of a simple laxative like a liquid petrolatum, when promoted by newspaper exploitation, is likely to be detrimental to health by overuse as well as by misuse.

The indirect advertisement to the public, which Rule 4 provides against, has been the means of inducing the extensive lay use of certain proprietaries. Naturally Rule 4 has been bitterly opposed by most proprietary firms. Arguing that many physicians dispense their own drugs, pharmaceutical firms have insisted that every medicinal preparation should bear on its label, not only the dose of the preparation, but also a statement of the diseases in which the article is indicated. Whether manufacturers anticipated the profession's resentment toward the claim that physicians determine the treatment and perhaps the diagnosis by means of the statements on labels, or because the Shirley amendment to the Food and Drugs Act makes the proprietor responsible for therapeutic claims on the label of a medicine, it is a fact that fewer preparations than formerly need to be refused for infringement on this rule. In fact, some thoroughly objectionable proprietaries make a show of being "ethical" by omitting all therapeutic discussion from the labels of their preparations.

*Therapeutic Claims.*—Rule 6 makes ineligible for "New and Nonofficial Remedies" any articles regarding which the manufacturer or his agents make unwarranted, exaggerated or misleading statements as to the therapeutic value. Recognizing the long established custom of therapeutic exaggeration, it has been most difficult to determine the degree of conservatism which might with fairness be required of a manufacturer. In view of the common acceptance of individual impressions as dependable evidence, it is often almost embarrassing to declare as incompetent the statement of some well-meaning and all-too-kind-hearted doctor. However, as the pitfalls of haphazard clinical trials become better known and the physician's mistrust of glowing accounts of marvelous cures more outspoken, the manufacturers' claims will be more moderate.

*Nomenclature.*—Were it possible to enact and enforce a law which would oblige manufacturers to sell their medicinal products under properly descriptive names and which would make it illegal for a physician to prescribe it unless he understood the meaning of such properly descriptive titles, then the Council might safely disband. In that case, physicians would discontinue the use of most proprietary preparations in favor of established drugs, and successful newcomers might each year be counted on the fingers of one hand. Such a rational nomenclature is not to be thought of, at least in our generation. Rule 8 requires that the name of an article shall not be misleading, that it shall not be therapeutically suggestive, and that established drugs shall not be disguised by fanciful titles. It recognizes the right of discoverers of new drugs to name their discoveries, and interposes no objection to arbitrary names for such products so long as such names are not misleading or do not suggest the therapeutic uses of the products. As the rule provides against the recognition of coined names for established nonproprietary drugs, so it requires that mixtures of drugs shall bear names descriptive of their composition. It would be a long step forward if physicians would recognize more fully the objections to the many proprietaries which have, as their only point of originality, a nondescriptive name for an old drug or a mixture of well known drugs. It is an encouraging sign that the Federal Trade Commission, when issuing licenses for the manufacture of synthetic drugs introduced under German patents, stipulated that all manufacturers authorized to make a given drug should use the same name for it.

*Irrational Articles.*—Rule 10 provides against the recognition of an article which, because of its composition, is useless or inimical to the best interests of the public and medical profession. This rule excludes medicaments which (1) are unessential modifications of established articles, or (2) are of no therapeutic value or (3) are irrational. With regard to the recognition of mixtures or compounds containing two or more active ingredients, the Council requires that the manufacturer establish the rationality of its combination. The rule has prevented the recognition of many unnecessary so-called ethical specialties. Though a mass of testimonials was often to be had for them, these contained no evidence that the mixture was superior to its potent ingredient, or that its therapeutic effect had been determined. That there is a healthy tendency to use single drugs for their definite action and to discard combinations (be they shotgun proprietaries or "mixed" vaccines) is perhaps best illustrated by the fact that at the last revision of the U. S. Pharmacopoeia a considerable number of complex antiquities were omitted from that book.

Feeling confident that this meets with the endorsement of the profession, the Council is examining more critically the evidence for the value of pharmaceutical mixtures.—Extracted from *Journal A. M. A.* of May 10.

